# A Neutral Risk on the Development of New-Onset Diabetes Mellitus (NODM) in Taiwanese Patients with Dyslipidaemia Treated with Fibrates

**DOI:** 10.1100/2012/392734

**Published:** 2012-07-31

**Authors:** Chien-Ying Lee, Kuang-Hua Huang, Chun-Che Lin, Tung-Han Tsai, Hung-Che Shih

**Affiliations:** ^1^Institute of Medicine, Chung Shan Medical University, Taichung 40201, Taiwan; ^2^Department of Pharmacy, Chung Shan Medical University Hospital, Taichung 40201, Taiwan; ^3^Department of Health Service Administration, College of Public Health, China Medical University, Taichung 40402, Taiwan; ^4^Division of Hepatogastroenterology, Department of Internal Medicine, Chung Shan Medical University Hospital, Taichung 40201, Taiwan; ^5^Department of Medicine, Chung Shan Medical University, Taichung 40201, Taiwan

## Abstract

There are no data on the incidence of new-onset diabetes mellitus (NODM ) in nondiabetic dyslipidaemia patients treated with fibrates. The aim of our study was to clarify these issues, to investigate the relationship between NODM and fibrate and whether the fibrates lead to increased risk for developing NODM. A retrospective cohort study was conducted by analyzing the Longitudinal Health Insurance Database (LHID 2005) of the National Health Insurance Research Database (NHIRD) from 2005 to 2010 to investigate all fibrate prescriptions for patients with dyslipidaemia. We estimated the hazard ratios (HRs) of NODM associated with fibrate use. We identified 145 NODM patients among 3,815 dyslipidaemic patients in the database for the study period. The risk estimates for NODM for users of fenofibrate (HR 1.30; 95% CI 0.82, 2.05) and gemfibrozil (HR 0.771; 95% CI 0.49, 1.22) were not associated with an increased risk of developing NODM (*P* > 0.05). Our results revealed that patients with dyslipidaemia who took fenofibrate and gemfibrozil had a neutral risk of NODM. The reasons may be associated with the fibrates have the properties that activate PPAR**α** and in some cases also activated PPAR**γ**, leading to showing a neutral risk of NODM.

## 1. Introduction

Many trials demonstrated the effectiveness of fibrates in improving dyslipidemia by lowering elevated plasma triglycerides, which are the most clinically used therapeutics in the treatment of hypertriglyceridemia. Several clinical trials have indicated a benefit of fibrate treatment in cardiovascular risk reduction and improvement of lipid profiles [1]. Furthermore, fibrate treatment decreased combined incidence of coronary heart disease events in primary and secondary prevention trials [1].

Statins are the first-choice agents for reducing LDL-C that significantly reduces cardiovascular events, these benefits apply to people with and without a history of diabetes mellitus (DM) [2, 3]. Individual statins differ in the extent to which they increase the risk of new-onset diabetes mellitus (NODM) [4]. Some studies showed that simvastatin, atorvastatin and rosuvastatin seem to have deleterious effects on glycaemic control [5–8], whereas pravastatin appears to have either neutral or beneficial effects [9, 10]. 

Three subtypes of PPAR (peroxisome proliferator-activated receptors) receptors, PPAR*α*, PPAR*γ*, and PPAR*δ*, have been recognized to date; their endogenous ligands include fatty acids and eicosanoids [11, 12]. Fibrates are structurally and pharmacologically related to the thiazolidinediones, a class of anti-diabetic drugs that also act on PPARs, more specifically PPAR*γ*. Fibrates activate PPAR, especially PPAR*α* [13]. Activating PPARs induces the transcription of a number of genes that facilitate lipid metabolism. This selective PPAR modulator concept may explain differences in biological activity [14]. 

Studies demonstrated that intensive-dose statin therapy was associated with a higher risk for NODM compared with moderate dosing, and the results confirmed a dose-dependent relationship for NODM [15]. There are no data on the incidence of NODM in nondiabetic dyslipidaemia patients treated with fibrates. The aim of our study was to investigate the relationship between NODM and whether the fibrates lead to increased risk for developing NODM. In this retrospective cohort study, we explored the effects of 2 different fibrates (fenofibrate and gemfibrozil) on the development of NODM in patients with dyslipidaemia. 

## 2. Materials and Methods

### 2.1. Study Design and Sample

A retrospective cohort study was conducted using the Longitudinal Health Insurance Database (LHID 2005) of the National Health Insurance Research Database (NHIRD) from 2005 to 2010 to investigate the fibrate prescriptions of patients with dyslipidaemia. We used the International Classification of Diseases, Ninth Revision (ICD-9) Clinical Modification code to define dyslipidaemia (ICD-9 codes 272), and diabetes mellitus (ICD-9 code 250).

**Figure 1:**
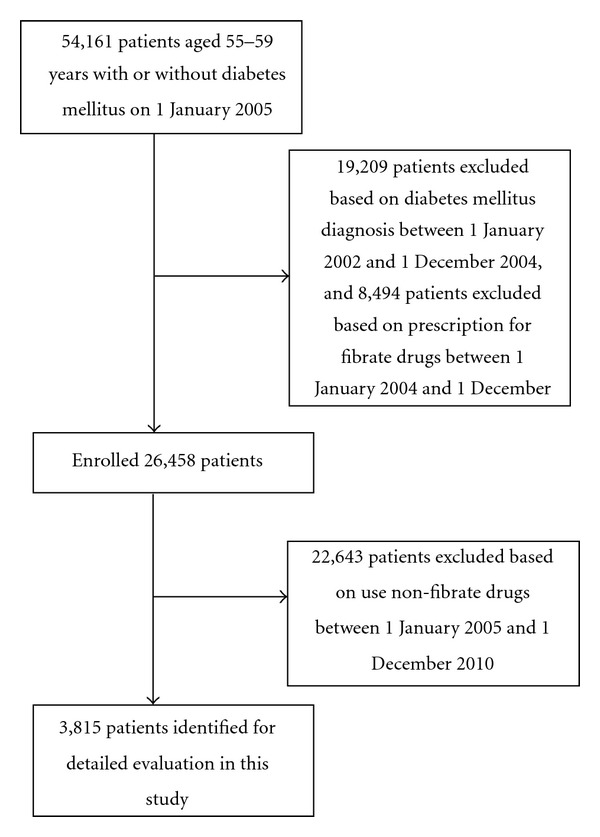
Flowchart of the selection of patients for inclusion.

This study initially included 54,161 patients that had dyslipidaemia without diabetes mellitus at baseline (1 January 2005), excluded 19,209 patients with diabetes mellitus from 2002 to 2004, and excluded another 8,494 patients that had used fibrates from 1 January 2004 to 1 December 2004. We identified subjects who received a diagnosis of NODM during the 5-year study period. Two types of fibrates (fenofibrate and gemfibrozil) were included for analysis. In Taiwan, these two drugs are available only by prescription. Patients who had used only one type of fibrate on a regular basis before the date NODM was diagnosed were categorized according to the fibrate type that they took. Of the remaining patients, 22,643 were excluded based on the use of nonfibrate drugs between 1 January 2005 and 1 December 2010. In January 2005, 3,815 nondiabetic dyslipidaemia outpatients (mean age, 55.59) were enrolled in the study. The primary endpoint of the study was NODM, which was defined as the first time that a diabetes code or prescription for antihyperglycemic drugs appeared in the outpatient claim records (Figure 1).

### 2.2. Statistical Analysis

Continuous variables are presented as mean ± SD. Differences in continuous variables were analysed using the unpaired Student's *t*-test. Categorical and discrete variables are presented as frequencies and percentages. When appropriate, they were compared by either Fisher's exact test or the chi-squared test. The effect of each of the two fibrates on the probability of developing NODM was corrected for by using the total exposure to each fibrate (expressed as total drug days). Time-to-event data were analysed using the log-rank test. Patients were removed from all time-to-event analyses at the time NODM was diagnosed or at death. In the univariate Cox regression model, the hazard ratio (HR) was used to estimate the incidence probability of developing NODM for each fibrate. Multiple Cox regression models were used to estimate the relationship between sex, age, medication, and mean dose of fibrates and development of NODM. All analyses were performed using SAS statistical analysis software, version 9.1 (SAS Institute Inc., Cary, NC, USA). A *P* value <0.05 was interpreted as being statistically significant.

## 3. Results

Of the 3,815 eligible participants, 145 (3.8%) developed NODM. There was no significant difference in age between the NODM and non-NODM patients; the mean ages in the female and male group were 56.10 and 55.57 years. Males comprised more than half (2,385 (62.52%)) of the sample population (Table 1).

Approximately 73.55% (2,806) of the patients took fenofibrate, and 26.45% (1,009) took gemfibrozil. No significant differences were observed in the mean doses of gemfibrozil prescribed between the groups; however, there was a significant difference in the mean doses of fenofibrate between the NODM and non-NODM patient groups (*P* < 0.05). (Table 1) For patients who used fenofibrate, the mean dose was 262.17 ± 238.50 in the NODM group and 222.03 ± 152.52 in the non-NODM group. Patients who used gemfibrozil, the mean dose is 703.90 ± 328.46 in NODM group and 637.44 ± 323.34 in non-NODM group (Table 1).

 Use of fenofibrate (HR 1.30; 95% CI 0.82, 2.05) and gemfibrozil (HR 0.771; 95% CI 0.49, 1.22) was not associated with an increased risk of developing NODM (*P* > 0.05) (Table 2). Similar results were obtained after controlling for age, sex, concomitant medication, and mean dose of each fibrate type (Table 2).

## 4. Discussion

In this retrospective longitudinal cohort study, we found that patients with dyslipidaemia who took fenofibrate and gemfibrozil were not associated with an increased risk of developing NODM and had a neutral risk of NODM. The possible reasons may be as follows. The PPAR nuclear receptor subfamily regulates a number of metabolic processes, including fatty acid *β*-oxidation, glucose utilization, cholesterol transport, and energy balance [16]. PPAR*α* and PPAR*γ* appear to play key roles in the catabolism and storage of fatty acids (FAs), a molecular mechanism whereby dietary lipids could affect overall energy balance and impact such metabolic diseases as obesity, atherosclerosis, and NIDDM [12], whereas PPAR*α* functions in lipid catabolism and homeostasis in the liver. PPAR*γ* has many activities, leading to complicated and even paradoxical effects on adipocyte biology, insulin action, cardiovascular disease, inflammation [17]. The PPAR*γ* subtype predominantly expressed in adipose tissue, appears to play a primary role in the storage of lipids in adipose tissue [12]. Activation of PPAR*γ* in mice and human is associated with a modest increase in plasma HDL-cholesterol and a decrease in plasma triglycerides [18]. Fibrates, PPAR*α* activator drugs which activate PPAR*α* and in some cases also activate PPAR*γ* [19, 20], demonstrate a broad spectrum of effects on lipids in man including reductions in total plasma cholesterol and triglyceride levels [20]. The antidiabetic thiazolidinediones (TZDs) are PPAR-*γ* activator drugs which selective PPAR*γ* ligands, enhance the actions of insulin in peripheral tissues, used in the treatment of type 2 diabetes mellitus to improve target cell insulin sensitivity. TZDs also reduce serum lipid levels in rodents and humans suffering from non-insulin-dependent diabetes mellitus (NIDDM) [21], which reduce plasma triglyceride, fatty acid, and insulin levels and increase HDL cholesterol levels [22, 23]. However, they have only a modest effect on dyslipidaemia, and they increase the fat mass and plasma volume [24]. We hypothesized that fibrates may have the properties that activate PPAR*α* and in some cases also activated PPAR*γ*, leading to showing a neutral risk of NODM. The patients included in our study may be not enough, further larger studies are necessary to clarify the issue of whether fibrates associated with NODM.

There was a significant difference in the mean doses of fenofibrate. We could find that the HR of fenofibrate was 1.30 (95% CI 0.82, 2.05), which tends to increase the risk of developing NODM, and *P* > 0.05 indicated that it was not associated with an increased risk of developing NODM. Gemfibrozil is a lipophilic compound, and about 95% of gemfibrozil is bound to serum albumin [25]. Fenofibrate also is a lipophilic compound, and it is highly protein bound (99%), primarily to albumin [26]. *In vitro *studies using human liver microsomes indicate that fenofibric acid is an inducer of CYP3A4, a weak inhibitor of CYP2C8, CYP2C19, and CYP2A6, and a mild-to-moderate inhibitor of CYP2C9 at therapeutic concentrations. Gemfibrozil is also an inducer of CYP3A4 but acts as both an inducer and an inhibitor of CYP2C8 [27]. Thus, these two most used fibrates (fenofibrate and gemfibrozil), although exerting similar beneficial actions on plasma lipids, may also exert distinct effects by yet unresolved mechanisms. Other influencing factors may be considered, including specific characteristics such as the formula of the fibrates, the subject's characteristics, such as race and body size, individuals with obesity, the metabolic syndrome, impaired fasting, glucose, or impaired glucose tolerance. Prospective studies are needed to clarify these issues regarding the relationship of other influencing factors with NODM and fibrates.

While FAs are essential biological components, elevated concentrations of circulating FAs are linked to a variety of disease states including obesity, atherosclerosis, and non-insulin-dependent diabetes mellitus (NIDDM). Thus, physiologic levels of FAs levels must be maintained within narrow limits [12]. Updated guidelines from the National Cholesterol Education Program Adult Treatment Panel III (NCEP ATP III) recognize the potential of statin–fibrate combination therapy in patients with mixed dyslipidemia and coronary heart disease (CHD) or CHD risk equivalents. It is well accepted that statins are the primary and more efficient method of reducing LDL-C levels even at low doses [28]. Fenofibrate has small or minimal effects on LDL-C levels, which depends on baseline TG levels [28]. Monotherapy for the treatment of dyslipidemias is commonly insufficient to achieve all lipid targets recommended by current guidelines. Therefore, the use of combined treatment has emerged as a new option in many cases in the last few years. The combination of fenofibrate with a statin, along with better improvements in lipid profile, has been shown to induce a marked increase in the ratio of large to small LDL subspecies compared with statin monotherapy [29]. Long-term, placebo-controlled trials with the combination of a fibrate and a statin with hard cardiovascular disease (CVD) outcomes are lacking evidence to support. Concomitant administration of fibrates with statin drugs increases the risk of muscle cramping, myopathy, and rhabdomyolysis. Combination of a statin with gemfibrozil appeared to cause more rhabdomyolysis than with newest fibrates (when compared with fenofibrate) [30]. Although some studies demonstrated that intensive-dose statin therapy was associated with a higher risk for NODM, from our study, we can realize that fibrates were not associated with an increased risk of developing NODM, leading to a neutral risk of NODM. However, a dose-dependent relationship between statins and NODM was confirmed. There is no reason to consider any changes with regard to short-term combination use of fenofibrate with a statin prescribing for those patients with mixed dyslipidemia and CHD or CHD risk equivalents, the cardiovascular benefits clearly outweigh the risk of developing diabetes. 

Some limitations of this study need to be emphasized. This was a descriptive, retrospective study conducted in Taiwan over a period of 5 years. Establishing cause and effect for each of the fibrates was not possible in this study. All cases in this study were collected from claim datasets of primary care clinics. Diagnoses (diagnostic codes: dyslipidaemia) were based on physician reporting only in Taiwan, and the risk factors for diabetes mellitus, such as obesity, family history, treatment adherence and diet, were not available in these secondary data. However, in the population-based data used here, we assumed that there were no differences among the two fibrate groups; therefore, it is not clear how our findings can be generalized to patients in different areas. Some of the apparent differences between the fibrates might potentially be explained by differences in total exposure to the different fibrates. Our data were taken from claim forms provided to the central regional branch of the BNHI in Taiwan from January 2002 to December 2010. Some of the apparent differences between the fibrates might potentially be explained by differences in total exposure to the different fibrates. Our data were taken from claim forms of the Bureau of National Health Insurance (BNHI) as a surrogate for drug exposure; the actual cholesterol levels that were achieved in the studied population were not known. However, physicians prescribed fibrates according to the BNHI guidelines in Taiwan.

## 5. Conclusions

Our results revealed that patients with dyslipidaemia who took fenofibrate and gemfibrozil were not associated with an increased risk of developing NODM and had a neutral risk of NODM. The reasons may be associated with the fact that fibrates have the properties that activate PPAR*α* and in some cases also activate PPAR*γ*, leading to showing a neutral risk of NODM. 

## Figures and Tables

**Table 1:** Descriptive characteristics of fibrate prescriptions in the population, 2005–2010.

Characteristics	NODM		No-NODM		Total		*P*-value
	*n*	%	*n*	%	*n*	%
Total	145	3.8	3,670	96.2	3,815	100	
Age (year, mean ± SD)	56.10 ± 12.21		55.57 ± 12.56		55.59 ± 12.54		0.616
Gender
Female	58	40.00	1,372	37.38	1,430	37.48	0.523
Male	87	60.00	2,298	62.62	2,385	62.52	
Drug group
Fenofibrate	104	71.72	2,702	73.62	2,806	73.55	0.611
Gemfibrozil	41	28.28	968	26.38	1,009	26.45	
Mean dose
Fenofibrate	262.17 ± 238.50		222.03 ± 152.52		223.52 ± 156.67		0.048
Gemfibrozil	703.90 ± 328.46		637.44 ± 323.34		640.14 ± 323.65		0.198

Differences in continuous variables were analysed using the unpaired student's *t*-test.

Categorical variables were analysed using by either Fisher's exact test or the chi-squared test.

**Table 2:** Cox analysis of fibrate prescriptions in the population, 2005–2010.

Variables	Unadjusted			Adjusted^a^		
	HR	95% C.I.	*P*-value	HR	95% C.I.	*P*-value
Fenofibrate	0.91	0.64–1.31	0.614	1.30	0.82–2.05	0.263
Gemfibrozil	1.10	0.76–1.58	0.614	0.771	0.49–1.22	0.263

^a^Adjusted for gender, age, and mean dose.

Multiple Cox regression models were used to estimate the relationship between sex, age, medication, and mean dose of fibrates and development of NODM.
